# Does Early Environmental Complexity Influence Tyrosine Hydroxylase in the Chicken Hippocampus and “Prefrontal” Caudolateral Nidopallium?

**DOI:** 10.3389/fvets.2016.00008

**Published:** 2016-02-11

**Authors:** Fernanda M. Tahamtani, Janicke Nordgreen, Margrethe Brantsæter, Gunn C. Østby, Rebecca E. Nordquist, Andrew M. Janczak

**Affiliations:** ^1^Animal Welfare Research Group, Department of Production Animal Clinical Sciences, School of Veterinary Sciences, Norwegian University of Life Sciences, Oslo, Norway; ^2^Emotion and Cognition Research Program, Department of Farm Animal Health, Utrecht University, Utrecht, Netherlands

**Keywords:** chicken, tyrosine hydroxylase, hippocampus, rearing, development, NCL, dopamine, brain lateralization

## Abstract

In adult chickens, the housing system influences hippocampal morphology and neurochemistry. However, no work has been done investigating the effects of the early life environment on chicken brain development. In the present study, we reared 67 commercial laying hens (*Gallus gallus domesticus*) in two environments that differed in the degree of complexity (aviary or cage system). These two groups were further divided into two age groups. At 20 weeks of age, 18 aviary-reared birds and 15 cage-reared birds were humanely euthanized and their brains dissected. At 24 weeks of age, a further 16 brains from aviary-reared birds and 18 brains from cage-reared birds were collected. These brains were prepared for immunohistochemical detection of tyrosine hydroxylase (TH), the rate-limiting enzyme in the biosynthesis of dopamine, in the hippocampus and the caudolateral nidopallium (NCL). There were no differences between the treatment groups in TH staining intensity in the hippocampus or the NCL. In the medial hippocampus, the right hemisphere had higher TH staining intensity compared to the left hemisphere. The opposite was true for the NCL, with the left hemisphere being more strongly stained compared to the right hemisphere. The present study supports the notion that the hippocampus is functionally lateralized, and our findings add to the body of knowledge on adult neural plasticity of the avian brain.

## Introduction

Environmental complexity and enrichment influence brain morphology and neurochemistry in mammals and birds (1), with enriched environments promoting brain development (2). Alterations in the brain as a response to environmental complexity have also been described in the fruit fly (*Drosophila melanogaster*), in fish and in crayfish [reviewed in Ref. (3)]. Even in adulthood, variations in housing environment can induce anatomical and chemical changes in the brain (4), something that was previously thought to happen only in the juvenile brain (3).

It was once believed that neural plasticity was limited to a critical period early in life (5). However, later research in the 70s and 80s showed that juvenile-young adult neural plasticity is not limited to a critical period but is triggered by experience and is independent of age (6, 7). In birds, hippocampal volume can be modified by experiences such as food storing (8) and migration (9). Once a minimal experience threshold is reached, hippocampal growth is triggered. On the other hand, continued deprivation of experience can result in attrition of hippocampal volume over time (7).

In the hippocampus and other brain areas involved in learning and memory, one of the key neurotransmitters is dopamine (10, 11). Therefore, effects of environmental experience on cognition may be due to changes in dopaminergic pathways, which are known to affect working memory in several species including species of birds (12), non-human primates (13, 14), and rodents (15). Dopamine D1 receptors in the prefrontal cortex are fundamental for the expression of brain-derived neurotrophic factor (BDNF), which is involved in synaptic plasticity and is essential for memory formation (16–18). Dopamine D1 receptor knockout mice have been shown to have spatial learning deficits (18, 19). Furthermore, administration of the D2 receptor antagonist haloperidol results in impaired reference and working memory in rats (20). Dopamine is also involved in reward processes and positive motivational affect, with studies showing that haloperidol suppresses both anticipatory and foraging behavior in chickens and in their wild ancestor, the Red Jungle fowl (*Gallus gallus*) (21, 22). In the prefrontal cortex, the application of D1 agonist increased the currents caused by *N*-methyl-d-aspartate (NMDA) receptors, one of the main components of the long-term potentiation (LTP) cascade, which is a major mechanism that underlies learning and memory (23).

The chicken (*G. gallus domesticus*) is a model organism for both basic and applied avian research (24). Chicks reared in pens with visual barriers have hippocampal neurons with longer dendrites compared to chicks reared without visual barriers (25). A study in adult laying hens has demonstrated that more environmentally complex housing conditions increase hippocampal cell soma size and result in a left skewed asymmetry in the density of tyrosine hydroxylase (TH), the rate-limiting enzyme in the biosynthesis of dopamine (2). However, it is not known whether the rearing environment experienced by chickens in the early stages of life affects the plasticity and development of the brain. Recent research indicates that rearing laying hens in a barren environment causes long-lasting deficits in spatial cognition, more specifically on working memory (26).

The aim of the present study was to test and describe the long-term impact of early environmental complexity on TH in the chicken hippocampus and the caudolateral nidopallium (NCL), the avian functional analog to the mammalian prefrontal cortex (10). We used commercial laying hens that were reared in two environments that differed in the level of complexity but were otherwise identical. One group of hens experienced the environmental complexity of an aviary rearing system, whereas the other group was reared in a cage rearing system. After the rearing period, both groups of hens were housed in custom-built pens for a period of 4 or 8 weeks. At the end of the housing period, hens from both rearing systems were humanely euthanized and their brains prepared for immunohistochemistry with an anti-TH antibody. We hypothesized that the two rearing systems would result in differences in TH staining in the hippocampus and NCL. We predicted that chickens reared in a barren environment of the cage rearing system would have reduced levels of TH in comparison to chickens reared in a complex aviary environment. Due to neural plasticity, one could also expect that the differences between the groups would decrease with time housed in an environment of intermediate complexity. To the best of our knowledge, no previous studies have investigated the effects of rearing environment on chicken neuroanatomy.

## Materials and Methods

### Subjects and Housing

Non-beak trimmed, female Dekalb white chickens (*G. gallus domesticus*) of ages 0–24 weeks and normal health status were used in this study. The rearing treatments were applied as described in Tahamtani et al. (26). The birds were hatched and reared in a single room in a Natura Primus 1600 system, designed for aviary rearing of laying pullets. The system consisted of cages stacked in three tiers on either side of a corridor. The house was 60 m × 20 m and housed 52,000 chickens. At delivery to the rearing farm, immediately after hatching, the birds were placed in cages in the first and second tiers of the aviary rows. No birds were housed on the third tier. At 4 weeks of age, half of the birds in the house were released from the aviary row by opening the cage doors and allowed to move freely through the corridor floor and each tier on either side of the corridor, until the end of the rearing phase at 16 weeks of age. Meanwhile, the other half of the birds were kept inside the cages. The aviary-reared and the cage-reared birds were housed in separate corridors throughout the rearing phase. Birds were in visual and auditory contact with one another within and between aviary corridors. Cage dimensions were 120 cm × 80 cm × 60 cm (length × height × width) (Housing type: Big Dutchman Natura Rearing). The density was 25 birds/m^2^ during the first 4 weeks of life for both treatments. After the cage doors were opened, the density of aviary-reared birds was reduced to 12 birds/m^2^ when taking account of the sum of floor space in aviary tiers and the hallway. All birds were exposed to the same light intensity, light schedule and temperatures, as recommended by the General Management Guide for Dekalb White Commercial Layer (27). Light and temperature were automatically controlled by a computer. Light was calibrated upon system installation using a lux measurer at hen height in various points of the house. Upon arrival, the temperature inside the rearing house was set to 32–29°C for the first week. The temperature was then gradually decreased until approximately 21°C at 5 weeks of age, at which point it was held constant until the end of the rearing period at 16 weeks of age. The birds were kept in a 4-h light/2-h dark light cycle at 20 lux for the first 7 days after arrival at the rearing farm. After 7 days, the light regime was adjusted to an 18-h light/6-h dark cycle. Subsequently, the light period was reduced by 2 h per week until 12 h of light was achieved at 5 weeks of age. At 2 weeks of age, the light intensity was gradually reduced from 20 to 5 lux at 5 weeks of age and kept at this level until the end of the rearing period at 16 weeks of age. Pullets were provided with *ad libitum* access to feed using a chain dispersal system and water. The feed type was conventional pullet feed produced and sold by Felleskjøpet, Norway. The diets used were “oppdrett 1” for 0–6-week-old birds, “avl egg 1” for 6–8-weeks-old birds and “oppdrett 2” for 8–17-week-old birds.

At 16 weeks of age, 80 birds from both housing systems, 40 per treatment, were transported to the poultry facilities at the Norwegian University of Life Sciences, campus Ås, Norway. The aviary birds were collected by the farmer from both the floor and the tiers. At the research facility, the birds were housed in custom-built pens in two adjacent rooms. The rooms housed in total 450 birds used in other parts of a larger project. The rooms were identical in size and shape and measured 5.90 m by 4.90 m. Each room contained 22 pens. The pens’ dimensions were 120 cm × 80 cm × 200 cm (length × width × height) and were built with wooden frames and chicken wire. Each pen contained one nest box, an elevated platform (80 cm × 50 cm) at 110 cm height and two perches, one at 70 cm height and one at 140 cm height. Feed was provided *ad libitum* using a feeder (50 cm in diameter) and water was provided *ad libitum* by nipple drinkers (two per pen). Each pen contained 12 birds. Birds were housed in mixed groups of six aviary-reared birds and six cage-reared birds per pen. All the birds were fitted with a transparent thin plastic ring around the right leg. The end of the ring was cut off at 90° (cage-reared birds) or at 45° (aviary-reared birds) to identify the treatment group to which they each belonged. No pecking behavior toward the plastic rings was observed. In addition, animal marker spray was used to ease the identification of each treatment group. The birds were sprayed with blue spray paint from wing to wing, or with dark green paint from between the shoulder blades to the tail. Both markings were balanced over treatment groups to preclude confounding treatment and color of the marking. This identification system was used in order to ensure that observers were blind to treatment. The facility operated on a light cycle that was altered according to recommendations by the Dekalb Management Guide (27). The temperature in the rooms was kept at 21–24°C. On arrival to the research facilities, the light was kept on for the first 24 h to allow the birds to find the feed and water. On the second day, the lighting schedule was set at 12-h light/12-h dark. Subsequently, the light period was increased by 30 min per week until 15 h of light per day was reached. The light intensity remained at 5 lux throughout the research period.

### Fixation and Sectioning

Brains were collected at 20 and 24 weeks of age, resulting in 20 brains collected per treatment, per age. Weeks 20 and 24 of age were chosen for brain collection in order to investigate changes in the brain soon after the end of the rearing period. Younger brains were not collected to avoid stress bias due to transport and transfer to research facilities at 16 weeks of age. Chickens were sedated using 0.5 ml/kg Zoletil mix (10 ml Rompun vet. (Xylazine 20 mg/ml), and 0.75 ml Butomidor (Butorphanol 10 mg/ml) mixed with one vial of Zoletil vet. powder (Tiletamine HCL 125 mg, and Zolazepam HCL 125 mg)) and humanely euthanized by cervical dislocation. Brains were removed whole and immersion fixed in 4% paraformaldehyde for 24 h at 4°C. They were then moved to a 30% sucrose solution (in PBS) at 4°C for 1 h, after which they were changed into a new 30% sucrose solution in PBS at 4°C for 48 h. Following this, brains were frozen by burying in dry ice for 3 min and transferred to a −20°C freezer. After 24 h, the brains were stored at −80°C.

**Figure 1:**
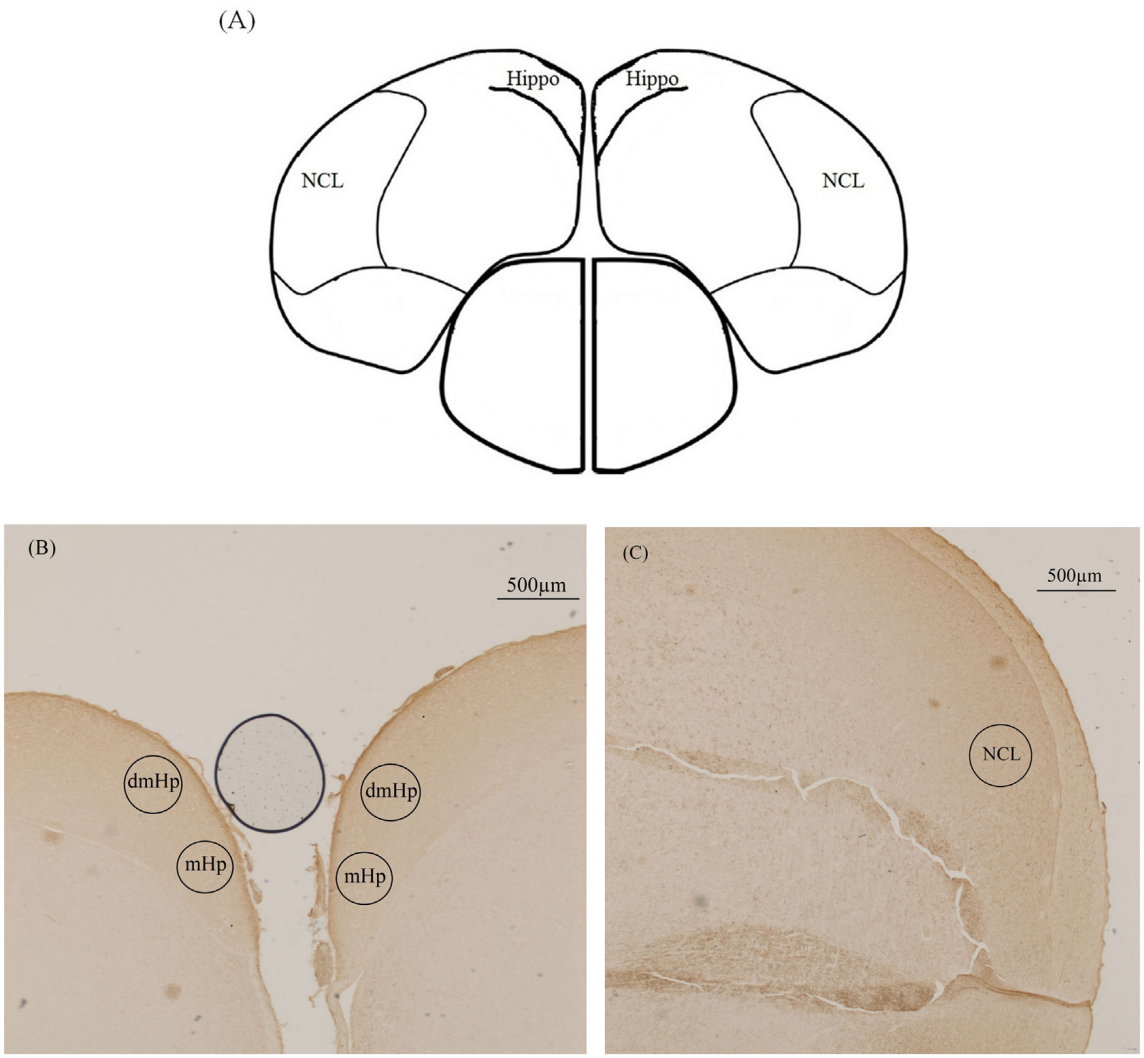
**Illustration of the location of the three analyzed brain areas in the immunohistochemical staining of tyrosine hydroxylase**. **(A)** Schematic drawing of the chicken brain sectioned along the coronal plane illustrating the hippocampus (Hippo) and caudolateral nidopallium (NCL) (interaural 2.56 mm). **(B)** Photograph of dorsomedial hippocampus (dmHp) and medial hippocampus (mHp). **(C)** Photograph of caudolateral nidopallium (NCL).

Brains were cryosectioned in the coronal plane (20 μm, Leica CM3050). Slices were collected in 10 parallel series and laid on Superfrost slides (Thermo Scientific, Braunschweig, Germany). A brain atlas for 2-week-old chickens was used to locate the regions of interest (ROI) (28) (Figure 1), taking into account the increased brain size for chickens at 20 and 24 weeks of age. Approximate area of sectioning corresponded with the appropriate figure numbers in the atlas: both the hippocampus (coded Hi1, Hi2, PHiM, PHil, PHil1 PHil2, and PHiA in the atlas) and NCL were sectioned from interaural 2.80–2.08 mm.

### Immunohistochemistry

Immunohistochemical detection of TH was performed in nine batches, with groups balanced across batches. Unless otherwise noted, all incubations and washes were performed at room temperature. The slides were washed three consecutive times for 5 min in 0.05M Tris Buffered Saline (TBS) and then incubated in 0.3% H_2_O_2_ in methanol for 10 min. The slides were then washed three times for 5 min in TBS with 0.05% Tween^®^20 (Sigma-Aldrich, Steinheim, Germany; TBS-T). Blocking was performed with 5% normal goat serum (NGS) in TBS-T for 60 min. Following blocking, the slides were incubated with rabbit polyclonal TH antibody (anti-TH, polyclonal rabbit IgG, Millipore, Amsterdam, the Netherlands; 1:250 in 1% NGS/TBS-T) overnight. Slides for negative control were included and incubated with 80 ng/ml of rabbit immunoglobulin fraction (normal; DAKO, Glostrup, Denmark). After three washes of 5 min in TBS-T, the slides were incubated with biotinylated goat anti-rabbit IgG (Vectastain ACB-Elite kit, Brunschwig Chemie B.V., Amsterdam, the Netherlands; 1:200 in 1% NGS/TBS-T) for 60 min in 37°C. Following this, the slides were washed three times for 5 min each in TBS-T and incubated in an avidin–biotin–peroxidase solution (Vectastain ACB-Elite kit, Brunschwig Chemie B.V., Amsterdam, the Netherlands; 1:100 in TBS) for 45 min. Following two washes of 5 min in TBS, the staining was visualized using 3′3-diaminobenzimin (DAB, Sigma-Aldrich, Chemie B.V., Zwijndrecht, the Netherlands). After rinsing in TBS, the slides were dehydrated in a graded alcohol series (70, 90, and 100%) followed by xylene (100%). Cover slips were mounted using DPX (Sigma-Aldrich, Steinheim, Germany).

### Quantification and Analysis

Slides were coded for treatment groups to allow blind analysis. Quantification of TH was performed as in Nordquist et al. (29). Quantification was done on both hemispheres. Hippocampal analysis was undertaken in two distinct areas, the medial hippocampus (mHp) and the dorsomedial hippocampus (dmHp) whereas, the NCL was analyzed as a whole. The ZEISS software ZEN Pro 2012, Blue edition, (ZEISS, Germany) was used for imaging with a Zeiss Imager M2 AX10 microscope and Zeiss Axiocam 506 color camera. ROI were selected under 10× magnification using the contours function, and the average pixel intensity in each ROI was calculated. Pixel intensity was measured for each area in each section, for both hemispheres. Pixel intensity is the intensity of pixels in the region of interest. The more white the image the higher is the intensity (see ZEISS software ZEN Pro 2012 http://www.zeiss.com/microscopy/en_de/products/microscope-software/zen.html#downloads). The background pixel intensity per section was also measured from an area of no staining in the section. The values from the ROIs were then corrected for variability in staining per section by subtracting these values from the background intensity. As intensity is on an inverted scale (high intensity levels mean low levels of staining), this background correction results in the difference between background intensity and staining intensity, a measure that is directly proportional to staining level. A total of five sections per brain per area of interest were quantified and averaged.

The effect of rearing environment on the TH staining intensity was tested in a repeated measures ANOVA, with brain ID as a random factor nested both in treatment and in the random factor pen, and treatment, room, age, and hemisphere as fixed factors. The interactions between treatment and age, age and hemisphere, treatment and hemisphere, and treatment age and hemisphere were also included in the model. The fixed factor room was found to be insignificant for all the brain areas analyzed and was, therefore, removed from the model. The statistical software used was JMP^®^11.1.1 (SAS Institute Inc.).

### Ethical Statement

This experimental work was approved by the Institutional Animal Care and Use Committee at NMBU under ID number 6190.

## Results

Due to technical difficulties in dissection and cryosectioning, some brains had to be excluded from the study resulting in a total sample size of 67 brains, 34 from aviary-reared birds (18 brains collected at 20 weeks of age from birds with the body weight: mean 1.62 kg ± 0.11 SD; and 16 brains collected at 24 weeks of age from birds with the body weight: mean 1.71 kg ± 0.12 SD) and 33 from cage-reared birds (15 brains collected at 20 weeks of age from birds with the body weight: mean 1.56 kg ± 0.08 SD; and 18 brains collected at 24 weeks of age from birds with the body weight: mean 1.67 ± 0.12 SD). Mean values for TH staining intensity in the mHp, dmHp, and NCL are presented in Table 1.

**Table 1:** **Mean and SD values for tyrosine hydroxylase staining intensity in the chicken mHp, dmHp, and NCL**.

Brain area	Hemisphere	Aviary		Cage	
		20 weeks	24 weeks	20 weeks	24 weeks
mHp	Right	1616.66 ± 521.51	1776.5 ± 515.75	1494.13 ± 507.96	1820.26 ± 575.64
	Left	1588.97 ± 532.1	1721.22 ± 521.84	1459.92 ± 472.42	1770.74 ± 558.02
dmHp	Right	1724.22 ± 408.85	1829.51 ± 422.82	1607.92 ± 406.89	1908.45 ± 459.25
	Left	1748.16 ± 426.73	1815.55 ± 431.76	1520.07 ± 385.15	1903.97 ± 481.9
NCL	Right	778.21 ± 166.10	642.80 ± 196.70	672.47 ± 184.70	679.95 ± 207.24
	Left	924.47 ± 229.0	845.74 ± 262.44	813.31 ± 217.80	928.77 ± 311.84

*The analyzed brains were of aviary and cage-reared chickens at 20 and 24 weeks of age*.

In general, no treatment effects were observed in the TH staining of the three brain areas studied. In the mHp, the right hemisphere of aviary and cage-reared birds of both ages had higher staining intensity for TH compared to the left hemisphere (*F*_1,63_ = 9.86; *p* = 0.003). There was also a tendency for brains at 24 weeks of age to have more TH compared to younger brains at 20 weeks of age (*F*_1,63_ = 3.25; *p* = 0.076). The results showed no effects of treatment or for any of the interactions between fixed factors on the staining intensity of TH in the mHp.

No effects were found on the pixel intensity of TH in the dmHP.

In the NCL, the results also indicated an effect of hemisphere on TH staining intensity, this time with the left hemisphere having higher levels (*F*_1,63_ = 74.04; *p* < 0.0001). In addition, there was an interaction effect between treatment and age (*F*_1,33,26_ = 7.36; *p* = 0 0.01). However, this effect was lost after *post hoc* testing (Tukey’s test *p* > 0.05). There was also a tendency of an interaction effect between age and hemisphere (*F*_1,63_ = 3.67; *p* = 0.06). The results showed no other effects of treatment, age, or interactions on TH staining intensity in the NCL.

## Discussion

Based on results reported in Tahamtani et al. (26) showing that cage-reared laying hens had worse working memory performance in a holeboard task compared to aviary-reared hens, it was hypothesized that these two rearing systems would result in differences in TH density in the hippocampus and the NCL. However, contrary to our predictions, we found no effect of aviary and cage rearing on the immunohistochemical staining intensity of TH in the hippocampus or the NCL. The chickens were reared in environments with varying levels of complexity until 16 weeks of age, at which point they were transported to experimental facilities and were housed in equal pens of intermediate complexity between aviary and cage systems. They remained in this type of housing until brain dissections at 20 and 24 weeks of age. It is possible that during the period of experimental housing, any differences between the rearing groups disappeared. It is also probable that an environment more complex than an aviary, such as a free-range system, could produce the expected differences in TH in laying hens (2). Also, differences in sexual maturation rates between the hens, particularly around the onset of lay, could have been a source of residual variation in the data. It is also worth noting that there might have been slight differences in light intensity in the different parts of the rearing house, particularly along the vertical plane. Aviary birds, free to move within the corridors, were likely exposed to more variable light conditions compared to birds that were enclosed in cages.

An alternative reason for the lack of rearing effects is that dopamine is not the sole modulator of cognitive function. A previous study found that adult hens in a free-range housing system had larger hippocampal cell soma sizes compared to hens housed in battery cages (2). Studies with 16-day-old chicks found that those reared with visual barriers had better spatial memory (30) and longer dendrites with more dendritic spines (25) compared to chicks reared without any barriers. Furthermore, memory formation and learning has been shown to be mediated by synaptic plasticity, LTP, and the receptors that regulate it [reviewed in Ref. (31)]. LTP, the long-lasting increase in synaptic efficiency induced by high-frequency stimulation, is dependent on NMDA receptors (32). The use of NMDA receptors antagonists (33) or NMDA knockout (34, 35) causes deficits in spatial memory. Therefore, it is possible that the effects of rearing environment on working memory seen in Tahamtani et al. (26) were due to changes in cell soma size, the NMDA receptors and/or LTP cascade rather than dopaminergic changes. Another possible explanation for the lack of rearing effects stems from the different inclusion criteria of the two studies. In Tahamtani et al. (26), hens included in the study were selected after a week of habituation training and any further hens that did not learn the task were excluded from analysis. In the present study, such procedures were not possible. It is likely, therefore, that the collection of brains studied here represents a wider range of intrinsic cognitive abilities.

The results did show, however, lateralization of TH staining intensity across brain hemispheres. For birds from both rearing treatments, the right mHp was more heavily stained compared to the left hemisphere. These results are in line with a growing body of evidence that the avian hippocampus is functionally and anatomically lateralized. Dendrites of the right hippocampus are longer and have more spines than the left hippocampus (25) and the encoding of the relative position of objects is done by the right hemisphere and not the left (36, 37). Interestingly, in a previous study, the left hippocampus of adult free-range hens was shown to have more TH-immunopositive fibers compared to the right (2), contradicting evidence for a right-skewed dominance of the avian hippocampus. Furthermore, the different results observed in the mHp and the dmHp suggest that these two areas may have different functions, despite both being part of the hippocampus. Future research should more closely investigate the effect of hippocampus area on the lateralization dominance of the avian hippocampus. Compared to the mHp, the opposite was observed in the NCL, with the left hemisphere having higher values for TH staining intensity than the right. The NCL is a specialized pallial area of the avian brain, which constitutes a functional equivalent to the mammalian prefrontal cortex (10) and has been shown to have a high density of NMDA receptors (12). The NCL is involved in decision making and learning (38) and pharmacological interventions such as the sodium channel blocker Tetrodotoxin and the NMDA receptor antagonist 2-amino-5-phosphonovalerianacid (APV) retard extinction learning (39, 40). Like the mammalian prefrontal cortex, the NCL has a low density of D1 receptors (12). Dopamine modulates higher cognitive functions in the prefrontal cortex, such as decision making and working memory, along an inverted-*U* (bell-shaped) curve where intermediate levels of dopamine/D1 activation are optimum [reviewed in Ref. (41)]. The results from the present study suggest a lateralization of TH in NCL hemispheres. This, in connection with previous literature, could indicate a differential role of NCL hemispheres in cognitive and executive functions.

In conclusion, we found no support for the hypothesis that varying exposure to environmental complexity during rearing should result in differences in TH staining in the hippocampus and NCL. However, the study did indicate that TH is lateralized in these brain areas.

## Author Contributions

FT: participated in the design of the study, collected samples, performed lab work, analyzed the data, and drafted the manuscript; JN: participated in the design of the study, collected samples, carried out data analysis, and drafted the manuscript; MB: participated in the design of the study, collected samples, and drafted the manuscript; GØ: performed lab work and drafted the manuscript; RN: participated in the design of the study, advised FT in lab analysis, and drafted the manuscript; AJ: led the project, participated in conception and design of the study, collected samples, and drafted the manuscript. All authors approved the final manuscript.

## Conflict of Interest Statement

No conflicts of interest exist in regards to this study. The funding organizations, the Foundation for Research Levy on Agricultural Products (FFL), the Agricultural Agreement Research Fund (JA), and Animalia (Norwegian Meat and Poultry Research Centre) finance applied agricultural research in collaboration with the private and public sectors. These parties’ sole interest in the present study was to support publication of unbiased results in order to provide advice to poultry rearers.
